# Assessing the FAIRness of Metabolic Bariatric Surgery Registries: a Comparative Analysis of Data Dictionaries from the UK, Germany, France, Netherlands, Norway, and Sweden

**DOI:** 10.1007/s11695-025-07701-2

**Published:** 2025-02-04

**Authors:** Bart Torensma, Mohamed Hany, Jodok M. Fink, Ahmed R. Ahmed, Ronald S. L. Liem, Andrea Lazzati, François Pattou, Johan Ottosson, Martijn G. Kersloot

**Affiliations:** 1 Leiden University Medical Center, Leiden, Netherlands; 2 Medical Research Institute, Alexandria University, Alexandria, Egypt; 3 Department of General and Visceral Surgery, Center for Surgery, Medical Center, University of Freiburg, Hugstetter Str. 55, Freiburg, Germany; 4 Imperial College London, London, UK; 5 Nederlandse Obesitas Kliniek West, Den Haag, Netherlands; 6 Groene Hart Ziekenhuis, Gouda, Netherlands; 7 Avicenne Hospital, Bobigny, France; 8 Centre Hospitalier Universitaire de Lille, Lille, France; 9 Pasteur Institute of Lille, Lille, France; 10 Department of Surgery, Faculty of Medicine and Health, Örebro University, Örebro, Sweden; 11 Amsterdam UMC location University of Amsterdam, Department of Medical Informatics, Meibergdreef 9, Amsterdam, Netherlands

**Keywords:** FAIR, Metabolic bariatric surgery, Registry, Data modeling

## Abstract

**Background:**

This study is part of an initiative to improve the FAIRness (Findability, Accessibility, Interoperability, Reusability) of metabolic bariatric surgery (MBS) registries globally. It explores the extent to which European registry data can be manually integrated without first making them FAIR and assesses these registries’ current level of FAIRness. The findings establish a baseline for evaluation and provide recommendations to enhance MBS data management practices.

**Methods:**

Data dictionaries from five national MBS registries in Germany, France, the Netherlands, the UK, and a combined registry for Scandinavia (Norway and Sweden) were evaluated regarding their ability to manually integrate registry datasets with one another. The FAIR Data Maturity Model from the Research Data Alliance (RDA) FAIR Data Maturity Model Working Group was used to assess the FAIRness of both metadata and data of the registries.

**Results:**

The registries showed significant variability in variables and coding structures, with inconsistent numerical formats and without linkage to international standards such as SNOMED CT, LOINC, or NCIt, making data integration labor-intensive and assumption-heavy. Despite the presence of data dictionaries, all registries failed the FAIR assessment because machine-readable data was unavailable, and only human-readable metadata was available in the form of data dictionaries in a spreadsheet.

**Conclusion:**

Our study reveals significant inconsistencies in data structuring and a failure to comply with the FAIR Principles, which limit effective data analysis and comparison. This emphasizes the critical need for standardized data management practices. We recommend four next steps to improve the FAIRness of MBS registries: (1) annotate data elements using standardized terminology systems, (2) deposit registry-level metadata in a repository, (3) request globally unique and persistent identifiers for datasets, and (4) define access restrictions.

## Introduction

Since 1975, global obesity rates have soared, with significant prevalence in the USA, Australia, the UK, the Pacific islands, and the Middle East, highlighting the urgency of research in obesity and related treatments [1–5]. Clinical registries are critical in identifying and evaluating disease epidemiology, treatment efficacy, and care quality. Combining data from multiple registries is an important step toward improving research on obesity and related treatments, as it creates a larger and more diverse dataset, increasing study power and generalizability and allowing for pattern detection. This yields more precise results and a better understanding of the factors contributing to obesity. To combine or integrate data from registries, standardized data collection and storage practices are essential. However, registries face challenges in standardizing data collection, as evidenced by Coulman et al.’s Delphi survey, which identified essential items for a metabolic bariatric surgery (MBS) Core Registry Set (CRS) but noted missing details for comprehensive research [6]. Akpinar et al. further identified disparities in registry data, with only a minor fraction of variables in agreement, emphasizing the need for standardized data practices [7].

**Fig. 1:**
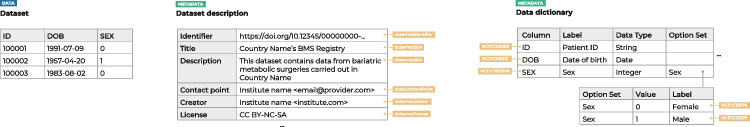
Example metadata from a fictitious dataset. Links to international standards offering unambiguous codes for variables and values and machine-readable descriptions of them are highlighted in orange

The introduction of the FAIR (Findable, Accessible, Interoperable, Reusable) Guiding Principles in 2016 marked a pivotal shift towards improving scientific data management [8]. These principles advocate for datasets to include clear descriptions (metadata) of the data and metadata on how and under what conditions the data can be accessed and reused. These descriptions should be readable for both humans (e.g., researchers), but most importantly, for machines (computers). An example of human- and machine-readable metadata is displayed in Fig. 1.

International standards for describing data in a machine-readable format include terminology systems such as SNOMED-CT (Systematized Nomenclature of Medicine-Clinical Terms) [9], LOINC (Logical Observation Identifiers Names and Codes) [10], and NCIt (the National Cancer Institute Thesaurus) [11]. These standards define unambiguous language-independent codes for variables and values, improving interoperability and data sharing across systems and institutions. This standardization is critical for automated data integration across multiple sources, managing increasing data volumes, and integrating emerging technologies such as AI and machine learning, allowing for advanced, privacy-preserving analytics of distributed datasets [12, 13], which are critical for advancing research and improving patient care in MBS. Moving to machine-readable metadata is, therefore, more than just a technical advance; it is also a necessary step toward utilizing these registries to their fullest extent, essential for promoting worldwide advances in patient care and MBS research. (Inter)national registries are increasingly adapting their data management processes and infrastructure to the FAIR Principles, with examples of registries for proton therapy research [14], rare disease research [15], and the quality of care in intensive care [16]. However, applying the FAIR Principles in MBS research still needs to be explored.

This study is part of a broader initiative to evaluate and enhance the FAIRness of MBS registries globally to perform analyses across multiple registry datasets. We emphasize that “FAIR data” does not necessarily mean “open data” and, therefore, explore federated learning as a solution to challenges posed by international data transfer laws. Our objective is to develop a FAIR data model that standardizes registry data, facilitating data exchange and integration across both national and international registries. By processing data locally and sharing only the results, this approach ensures compliance with legal and ethical standards while aligning with the FAIR Principles. Importantly, by making data FAIR (which does not necessarily imply opening it), we aim to enable federated analysis and learning.

This study is the first phase of the initiative, in which we evaluate (1) to what extent data from five European registries can be manually integrated without FAIRification and (2) the current state of FAIR (FAIRness) of these registries. We aim to establish a baseline FAIRness assessment and provide recommendations for MBS data management practices, to test the integration of data from multiple registries to enhance research quality and outcomes.

## Materials and Methods

Our approach consisted of two key steps: first, we evaluated the harmonization of different data dictionaries and manually tested the feasibility of integrating registry datasets; second, we assessed adherence to each FAIR principle (FAIRness) using a set of established criteria and indicators.

### Used Data Dictionaries and Datasets

Data dictionaries and datasets were requested from five MBS registries located in Germany, France, The Netherlands, and the UK and a combined registry for Scandinavia, with data from Norway and Sweden. Either a co-author who coordinated one of the registries or co-authors who were in contact with these registries purposively/conveniently sampled these specific registries.

### Evaluation of Manual Data Integration

We evaluated the harmonization of the data dictionaries and datasets to test manual data integration across the registries. This included assessing variable naming, coding values (e.g., Male as 0 for categorical variable Sex), numeric formats, and investigating the domains represented in the registries. To process the data dictionaries, variable names were translated to English using DeepL. R (version 4.0.4, utilizing dplyr, tidyr, magrittr, readr, purrr, stringr, forcats, lubridate, and tibble) was used to perform the manual integration of the registry datasets.

### Evaluation of FAIRness

The FAIR Data Maturity Model from the Research Data Alliance (RDA) FAIR Data Maturity Model Working Group was used to assess the FAIRness of both metadata and data of the registries [17]. This model consists of 41 indicators that help gain insight into the current FAIRness of the registries and the aspects that can be improved to increase the potential for the reuse of research data. Each indicator was linked to a FAIR Principle and was assigned a priority on a 3-point scale [17]. This scale categorizes indicators based on their significance in achieving FAIRness: “Essential” indicators are crucial and mandatory for FAIRness, “Important” indicators substantially enhance FAIRness but are not always critical, and “Useful” indicators are beneficial but less important. For every registry, the indicators were evaluated using a binary pass-or-fail scale, determining whether the indicators’ criteria were fulfilled. Important to note is that the FAIR Principles focus on Findability, Accessibility, Interoperability, and Reusability both for humans and machines, with specific emphasis on machine-actionability (i.e., the “machine knows what I mean” [18] (Appendix).

## Results

The variables in each registry varied, reflecting the diverse setup of the registries and data collection practices in MBS. An overview of the number of variables and used languages, naming conventions, and international standards can be found in Table 1. Due to the different languages used and limited information in data dictionaries, harmonizing or normalizing variables measuring the same concept was impossible. However, common domains could be identified across the registries. These domains included (1) patient characteristics (basic demographic and clinical data), (2) comorbidities (data on additional medical conditions present), (3) screening/pre-operative assessment (data on pre-surgery evaluations), (4) medical history (data on patient’s past medical events and conditions), (5) procedure information (data about undertaken bariatric surgery procedures), (6) complications (data on post-surgical complications, if any), and (7) lab (data on performed laboratory tests).

**Table 1:** Summary of the registries’ number of variables and used languages, naming conventions, and the use of international standards in the data dictionary

	Variables (*n*)	Language	Naming convention	International standards
The Netherlands	304	Dutch	Solely lowercase letters, without spaces	*None*
Scandinavia (Norway & Sweden)	903	Swedish, Norwegian, and English	Mixed capitalization, with spaces	*None*
Germany	1000	German	Mixed capitalization, with spaces	*None*
France	192	French	Mixed capitalization, with spaces	*None*
UK	64	English	Mixed capitalization, without spaces	*None*

### Evaluation of Manual Data Integration

Even though all registries shared the same domains, the number of variables and coding structures varied greatly. Numerical formats (dot, comma, units) were inconsistent, and none of the data dictionaries or coded values were linked to international standards such as SNOMED CT, LOINC, or NCIt. This lack of standardization meant there were no unambiguous definitions of the variables and coded values. Translating the variable names and coded values into English and re-coding values made the process of integrating data labor-intensive. In addition, it required many assumptions about the collected variables and their values and units, as identical variable names did not necessarily imply the same type of measured concept, nor the same unit, or time (e.g., systolic vs. diastolic blood pressure, length in meters vs. centimeters, weight measured at baseline vs. at follow-up). The lack of uniformity and standardization in data handling practices prevented us from combining the datasets and perform a comprehensive comparative analysis across them.

### Evaluation of FAIRness

All dictionaries were evaluated using the 41 FAIR Data Maturity Model items. Our assessment revealed that solely human-readable metadata was available for the registries as data dictionaries in a spreadsheet. Despite having data dictionaries in place, all registries failed the FAIR assessment, primarily because the existing metadata is only readable by other researchers (humans), requires translation and assumption-making, and cannot be automatically processed by computers (machines). The questions about manual access to the (meta)data were not answered, as we could only obtain data dictionaries via email and we had no way of accessing the data and metadata in another way. The full evaluation is shown in Table 2.

**Table 2:** FAIR assessment of the registries of France (FR), Germany (DE), Netherlands (NL), Scandinavia (SC), and the UK

Principle	Indicator	Priority	FR	DE	NL	SC	UK
F1	01 M: Metadata is identified by a persistent identifier	■■■	✕	✕	✕	✕	✕
F1	01D: Data is identified by a persistent identifier	■■■	✕	✕	✕	✕	✕
F1	02 M: Metadata is identified by a globally unique identifier	■■■	✕	✕	✕	✕	✕
F1	02D: Data is identified by a globally unique identifier	■■■	✕	✕	✕	✕	✕
F2	01 M: Rich metadata is provided to allow discovery	■■■	✕	✕	✕	✕	✕
F3	01 M: Metadata includes the identifier for the data	■■■	✕	✕	✕	✕	✕
F4	01 M: Metadata is offered in such a way that it can be harvested and indexed	■■■	✕	✕	✕	✕	✕
A1	01 M: Metadata contains information to enable the user to get access to the data	■■□	✕	✕	✕	✕	✕
A1	02 M: Metadata can be accessed manually (i.e., with human intervention)	■■■	~	~	~	~	~
A1	01D: Data can be accessed manually (i.e., with human intervention)	■■■	~	~	~	~	~
A1	03 M: Metadata identifier resolves to a metadata record	■■■	✕	✕	✕	✕	✕
A1	03D: Data identifier resolves to a digital object	■■■	✕	✕	✕	✕	✕
A1	04 M: Metadata is accessed through standardized protocol	■■■	✕	✕	✕	✕	✕
A1	04D: Data is accessible through standardized protocol	■■■	✕	✕	✕	✕	✕
A1	05D: Data can be accessed automatically (i.e., by a computer program)	■■□	✕	✕	✕	✕	✕
A1.1	01 M: Metadata is accessible through a free access protocol	■■■	✕	✕	✕	✕	✕
A1.1	01D: Data is accessible through a free access protocol	■■□	✕	✕	✕	✕	✕
A1.2	01D: Data is accessible through an access protocol that supports authentication and authorisation	■□□	✕	✕	✕	✕	✕
A2	01 M: Metadata is guaranteed to remain available after data is no longer available	■■■	✕	✕	✕	✕	✕
I1	01 M: Metadata uses knowledge representation expressed in standardized format	■■□	✕	✕	✕	✕	✕
I1	01D: Data uses knowledge representation expressed in standardized format	■■□	✕	✕	✕	✕	✕
I1	02 M: Metadata uses machine-understandable knowledge representation	■■□	✕	✕	✕	✕	✕
I1	02D: Data uses machine-understandable knowledge representation	■■□	✕	✕	✕	✕	✕
I2	01 M: Metadata uses FAIR-compliant vocabularies	■■□	✕	✕	✕	✕	✕
I2	01D: Data uses FAIR-compliant vocabularies	■□□	✕	✕	✕	✕	✕
I3	01 M: Metadata includes references to other metadata	■■□	✕	✕	✕	✕	✕
I3	01D: Data includes references to other data	■□□	✕	✕	✕	✕	✕
I3	02 M: Metadata includes references to other data	■□□	✕	✕	✕	✕	✕
I3	02D: Data includes qualified references to other data	■□□	✕	✕	✕	✕	✕
I3	03 M: Metadata includes qualified references to other metadata	■■□	✕	✕	✕	✕	✕
I3	04 M: Metadata include qualified references to other data	■□□	✕	✕	✕	✕	✕
R1	01 M: Plurality of accurate and relevant attributes are provided to allow reuse	■■■	~	~	~	~	~
R1.1	01 M: Metadata includes information about the license under which the data can be reused	■■■	✕	✕	✕	✕	✕
R1.1	02 M: Metadata refers to a standard reuse license	■■□	✕	✕	✕	✕	✕
R1.1	03 M: Metadata refers to a machine-understandable reuse license	■■□	✕	✕	✕	✕	✕
R1.2	01 M: Metadata includes provenance information according to community-specific standards	■■□	✕	✕	✕	✕	✕
R1.2	02 M: Metadata includes provenance information according to a cross-community language	■□□	✕	✕	✕	✕	✕
R1.3	01 M: Metadata complies with a community standard	■■■	✕	✕	✕	✕	✕
R1.3	01D: Data complies with a community standard	■■■	✕	✕	✕	✕	✕
R1.3	02 M: Metadata is expressed in compliance with a machine-understandable community standard	■■■	✕	✕	✕	✕	✕
R1.3	02D: Data is expressed in compliance with a machine-understandable community standard	■■□	✕	✕	✕	✕	✕

Priority: ■■■ Essential, ■■□ Important, ■□□ Useful

## Discussion

Our analysis of data dictionaries and datasets from five national European registries revealed significant barriers to data interoperability and a notable lack of adherence to the FAIR Principles. The registries’ variables and coding structures varied greatly, with inconsistent numerical formats and no reference to international standards such as SNOMED CT, LOINC, or NCIt. This made data integration labor-intensive and assumption-heavy and ultimately hamper the ability to combine datasets for comprehensive analysis and limiting the potential for cross-registry studies, post-intervention surveillance, and embedded randomized clinical trials. These findings underscore the critical need for the implementation of the FAIR Principles in clinical registries, in particular enhanced data and metadata standardization, to advance MBS research and ensure reliable outcomes.

However, to put our findings in perspective, the FAIR Principles are still in the early stages of adoption in various specialties, including medical and non-medical fields [19]. For many researchers, it is still unclear how data can be made FAIR for both humans and machines [20, 21]. While the potential benefits of making data FAIR are acknowledged, concrete examples of their implementation and outcomes still need to be improved. This provides an opportunity for the field of MBS to take the lead in adopting the FAIR Principles, potentially catalyzing broader acceptance and application across disciplines to improve research and clinical care efficiency.

Furthermore, our analysis of data dictionaries of national registries revealed that, despite their shortcomings in adhering to the FAIR Principles, each national registry has made commendable efforts in establishing and maintaining databases. These databases have been critical in systematically collecting and documenting data, contributing valuable insights into surgical outcomes, patient safety, and treatment efficacy in the MBS field. Enhancing their FAIRness will ultimately promote a more collaborative and effective research ecosystem and maximize their impact on global health outcomes in MBS.

### Lack of Standardization and Machine-Readable (Meta)Data

The results of our efforts to manually integrate data from the various registries and the results from the FAIR assessment indicate a significant opportunity for improvement in these registries’ data handling practices. Current practices do not focus on making the collected (meta)data FAIR for both humans and machines. Instead, all registries concentrate solely on human-readable metadata in spreadsheet-based data dictionaries. While having these metadata is a step in the right direction, the absence of machine-readable metadata imposes significant constraints. To enable integration of data and large-scale data analysis, it is essential that the data includes clear, unambiguous, and standardized descriptions for each variable collected. Unlike researchers, machines cannot infer details about the data (e.g., if the variable “weight” lacks a unit of measure, one might assume it is in kilograms based on the EU context and data range). Furthermore, relying on researchers to make these assumptions can introduce errors and inconsistencies, as different researchers might interpret the data differently. This subjective interpretation undermines the reliability and reproducibility of the analysis, highlighting the importance of providing comprehensive metadata to ensure accurate and consistent understanding across both humans and machines.

### Recommendations for “FAIRer” MBS Registries

To unlock the full potential of the data and advance research and patient care in this field, it is critical that MBS registries adhere to the FAIR Principles and for this mainly focus more on the machine-readability aspect [22]. Based on the essential indicators from the leveraged FAIR Data Maturity Model, we recommend four next steps for improving the FAIRness of the registries (Box 1).

Box 1. Four next steps for improving the FAIRness of MBS registries. FAIR Data Maturity Model indicators are referenced between brackets

**Table:** 

1. Annotate data elements using standardized terminology systems
• Ensure that descriptions of data elements are consistent throughout the dataset, specifying what is collected and how it is collected. This includes linking to definitions in terminology systems (such as SNOMED CT, LOINC, and NCIt) for each data element. (R1.3-01 M, R1.3-01D, R1.3-02 M)
- This includes units of measurement and coded options (for example, “Male” for the variable “Sex”) using these terminology systems
2. Deposit registry-level metadata in a repository
• Create and store metadata describing the registry in both human and machine-readable formats. Ensure that this metadata complies with domain standards such as DCAT and DataCite. (F2-01 M, R1-01 M, R1.3-01 M, R1.3-02 M)
• Deposit the metadata in a trusted repository, such as FAIRsharing to ensure that it is discoverable by others. This repository should allow for automated metadata browsing, thereby facilitating accessibility and reuse. (F4-01 M, A1-02 M, A1-03 M, A1-04 M, A1.1-01 M) [23]
• Register a DOI (Digital Object Identifier, by depositing the metadata) for the metadata to provide a persistent identifier. This step is crucial for the long-term traceability and citability of the registry and its (meta)data. (F1-01 M, F1-02 M)
• Verify that the repository maintains the metadata even if the underlying data becomes unavailable. This ensures that the registry’s information remains accessible over time. (A2-01 M)
3. Request globally unique and persistent identifiers for datasets
• Register a DOI for each dataset within the registry as well. (F1-01D, F1-02D)
• Due to legal and ethical considerations it would not be possible to make the data of the registries publicly available. However, linking the identifier to the metadata enables the dataset to be cited and referenced in future research, even if access is restricted. (A1-03D, A1-04D, F3-01 M)
4. Define access conditions
• Clearly define who can access and reuse the data, as well as the conditions under which access is granted, and include this information in the metadata. Providing this information in both human- and machine-readable formats ensures transparency and facilitates requests for data access. Even a basic mention of how to request access would be a significant improvement over current registry practices. (A1-02D, R1-01 M, R1.1-01 M) [9–11]

After improving the registries’ FAIRness by following these recommendations, the registry (meta)data can be more easily found and reused. The use of unambiguous data definitions from terminology systems allows for more efficient data integration. Once integrated, analyses can be performed by recoding the unified data definitions (e.g., converting a SNOMED CT concept ID such as 248153007 into binary codes preferred by statisticians, such as 1 or 0). These recoding scripts can be reused across different registries, alongside standard analysis scripts (e.g., regression analyses and predictive modeling), which helps save time during the pre-processing and analysis stages.

The MBS community and data managers must prioritize foundational aspects of data management in alignment with the FAIR principles. This begins with ensuring that data and variables adhere to standardized terminologies, such as SNOMED codes, which enhance both interoperability and reusability. The importance of establishing these standardized structures cannot be overstated, as it forms the basis for seamless data integration across different registries and institutions.

Once data is made FAIR, the next step is to develop scripts that convert standardized variables into formats suitable for statistical analysis. This preprocessing is essential not only for ensuring that exploratory data analysis (EDA) can be performed, but also for allowing advanced techniques, such as regression analyses, machine learning, and predictive modeling, to be applied effectively. Additionally, these preprocessing scripts can be reused across studies, further enhancing the efficiency and reproducibility of MBS research.

While these steps are essential, challenges remain. For example, many MBS registries might not have the resources to immediately implement these changes. This reinforces the importance of collaboration within the MBS community to share tools, scripts, and best practices. The field can draw inspiration from other medical areas, such as oncology or cardiovascular research, where the application of the FAIR principles has started to show success in integrating large datasets across institutions.

The key message for the MBS community is that, after identifying critical points in data management, the focus should be on making data and variables FAIR. Doing so will lay a solid foundation for merging and transforming data into analyzable formats, ultimately enabling comprehensive and advanced statistical analyses, which are widely used in epidemiology and data science. This approach will lead to more reliable and actionable insights in both clinical and epidemiological research.

## Future Studies

As previously stated, this study is part of a multi-phase project to increase the FAIRness of MBS registries. Following this study, we will proceed to phase 2, which will include a semi-automated systematic review of PubMed articles to identify frequently collected variables in our field. In phase 3, we will use insights from the current study (phase 1) and frequently collected variables (phase 2) to create a (semantic) data model based on the previous two phases. This model will improve data sharing and interoperability, resulting in greater research efficiency and collaboration. Both phases aim to advance scientific knowledge and data management practices significantly.

### Limitations

A limitation of our study is the limited prior research on the practical implementation of the FAIR Principles in medical research, particularly in the MBS field. This indicates that our subject is novel, but it also makes it challenging to determine precisely what registry data management enhancements are required to make it the data more FAIR [22]. It is, therefore, challenging to make firm recommendations for improvement, as there are yet to be any clear standards or guidelines in place. We aim to develop more understanding and guidance in phases 2 and 3.

## Conclusion

Our study highlights significant data structuring inconsistencies and a need to implement FAIR Principles in European MBS registries. These issues impede effective data comparison and analysis, emphasizing the critical need for standardized data management practices. Given the novelty of applying the FAIR Principles to medical research, particularly MBS, future efforts should focus on developing clear guidelines for implementing the FAIR Principles to advance MBS research and improve patient care. We have recommended four next steps to strengthen the FAIRness of MBS registries, and we intend to expand on these recommendations in future phases of the project.

## Data Availability

No datasets were generated or analysed during the current study.

## Appendix Summary of the FAIR Data Principles

- **Findability** means that the data can be discovered by both humans and machines, for instance by exposing meaningful machine-actionable metadata and keywords to search engines and research data catalogs. The data are referenced with unique and persistent identifiers (e.g. DOIs or Handles) and the metadata include the identifier of the data they describe.
- **Accessible** means that the data are archived in long-term storage and can be made available using standard technical procedures. This does not mean that the data have to be openly available for everyone, but information on how the data could be retrieved (or not) has to be available. For example, data can be marked “Access only with explicit permission from the author” and include the author’s contact details. Ideally, though, the information about data accessibility can also be read by machines, e.g. by way of machine-readable standard licenses.
- **Interoperable** means that the data can be exchanged and used across different applications and systems — also in the future, for example, by using open file formats. It also means that the data can be integrated with other data from the same research field or data from other research fields. This is made possible by using metadata standards, standard ontologies, and controlled vocabularies as well as meaningful links between the data and related digital research objects.
- **Reusable** means that the data are well documented and curated and provide rich information about the context of data creation. The data should conform to community standards and include clear terms and conditions on how the data may be accessed and reused, preferably by applying machine-readable standard licenses. This allows others either to assess and validate the results of the original study, thus ensuring data reproducibility, or to design new projects based on the original results, in other words data reuse in the stricter sense. Reusable data encourages collaboration and avoids duplication of effort.

## References

[1] WHO. https://www.who.int/news-room/fact-sheets/detail/obesity-and-overweight. Accessed 23 Sept 2024.

[2] Bryan Stierman, Joseph Afful, Margaret D. Carroll, Te-Ching Chen. National Health Statistics Reports, Number 158, June 14, 2021. 2021. 10.15620/cdc:106273 PMC11513744 39380201

[3] Moody A. Health survey for England 2019: overweight and obesity in adults and children. https://digital.nhs.uk/data-and-information/publications/statistical/health-survey-for-england/2019. Accessed 23 Sept 2024.

[4] Australian Institute of Health and Welfare. Overweight and obesity. 2022. https://www.aihw.gov.au/repor ts/australias-health/overweight-and-obesity Accessed 24 Jan 2023. 2022.

[5] Abarca-Gómez L, Abdeen ZA, Hamid ZA, et al. Worldwide trends in body-mass index, underweight, overweight, and obesity from 1975 to 2016: a pooled analysis of 2416 population-based measurement studies in 128·9 million children, adolescents, and adults. The Lancet. 2017;390:2627–42. 10.1016/S0140-6736(17)32129-3 PMC5735219 29029897

[6] Coulman KD, Chalmers K, Blazeby J, et al. Development of a bariatric surgery core data set for an international registry. Obes Surg. 2023;33:1463–75. 36959437 10.1007/s11695-023-06545-y PMC10156789

[7] Akpinar EO, Marang- Van De Mheen PJ, Nienhuijs SW, et al. National bariatric surgery registries: an international comparison. Obes Surg. 2021;31:3031–9. 33786743 10.1007/s11695-021-05359-0 PMC8175300

[8] Wilkinson MD, Dumontier M, Aalbersberg I, et al. The FAIR guiding principles for scientific data management and stewardship. Sci Data. 2016;3:160018. 26978244 10.1038/sdata.2016.18 PMC4792175

[9] SNOMED international. https://www.snomed.org. Accessed 23 Sept 2024.

[10] LOINC (Logical Observation Identifiers Names and Codes). https://loinc.org. Accessed 23 Sept 2024.

[11] NCI Thesaurus. Available from: https://ncithesaurus.nci.nih.gov/ncitbrowser/. Accessed 23 Sept 2024.

[12] Moreira JLR, Bonino L, Ferreira Pires L, et al. Towards findable, accessible, interoperable and reusable (FAIR) data repositories: improving a data repository to behave as a FAIR data point | Repositórios para dados localizáveis, acessíveis, interoperáveis e reutilizáveis (FAIR): adaptando um repositório de dados para se comportar como um FAIR Data Point. Liinc Rev [Internet]. 2019 [cited 2024 Mar 12];15. Available from: http://revista.ibict.br/liinc/article/view/4817.

[13] Choudhury A, van Soest J, Nayak S, Dekker A. Personal health train on FHIR: a privacy preserving federated approach for analyzing FAIR data in healthcare. In: Bhattacharjee A, Borgohain SKr, Soni B, Verma G, Gao X-Z, editors. Machine Learning, Image Processing, Network Security and Data Sciences. Singapore: Springer Singapore; 2020. p. 85–95.

[14] Sloep M, Kalendralis P, Choudhury A, et al. A knowledge graph representation of baseline characteristics for the Dutch proton therapy research registry. Clin Transl Radiation Oncol. 2021;31:93–6. 10.1016/j.ctro.2021.10.001 PMC8505268 34667884

[15] Kaliyaperumal R, Wilkinson MD, Moreno PA, et al. Semantic modelling of common data elements for rare disease registries, and a prototype workflow for their deployment over registry data. J Biomed Semant. 2022;13:9. 10.1186/s13326-022-00264-6 PMC8922780 35292119

[16] Puttmann D, De Keizer N, Cornet R, et al. FAIRifying a quality registry using OMOP CDM: challenges and solutions. In: Séroussi B, Weber P, Dhombres F, Grouin C, Liebe J-D, Pelayo S, et al., editors. Studies in Health Technology and Informatics [Internet]. IOS Press; 2022 [cited 2024 Jun 14]. Available from: 10.3233/SHTI220476 10.3233/SHTI220476 35612098

[17] Bahim C, Casorrán-Amilburu C, Dekkers M, et al. The FAIR data maturity model: an approach to harmonise FAIR assessments. Data Sci J. 2020;19:41.

[18] Mons B, Schultes E, Liu F, et al. The FAIR principles: first generation implementation choices and challenges. Data Intellegence. 2020;2:1–9.

[19] Van Reisen M, Stokmans M, Basajja M, et al. Towards the tipping point for FAIR implementation. Data Intellegence. 2020;2:264–75.

[20] Kersloot MG, Abu-Hanna A, Cornet R, et al. Perceptions and behavior of clinical researchers and research support staff regarding data FAIRification. Sci Data. 2022;9:241. 35624282 10.1038/s41597-022-01325-2 PMC9142513

[21] Mons B, Neylon C, Velterop J, et al. Cloudy, increasingly FAIR; revisiting the FAIR Data guiding principles for the European Open Science Cloud. ISU. 2017;37:49–56.

[22] Kersloot MG, Jacobsen A, Groenen KHJ, et al. De-novo FAIRification via an electronic data capture system by automated transformation of filled electronic Case Report Forms into machine-readable data. J Biomed Inform. 2021;122:103897. 34454078 10.1016/j.jbi.2021.103897

[23] A curated, informative and educational resource on data and metadata standards, inter-related to databases and data policies. https://fairsharing.org. Accessed 23 Sept 2024.

